# MONITORING CHRONIC PAIN AND QUALITY OF LIFE USING TABLET SURVEYS IN OLDER ADULTS LIVING IN RURAL ARKANSAS

**DOI:** 10.1093/geroni/igae098.4025

**Published:** 2024-12-31

**Authors:** Regina Gibson, Gohar Azhar, Shakshi Sharma, Riley Swafford, Karen Coker, Jeanne Y Wei

**Affiliations:** University of Arkansas for Medical Sciences, Little Rock, Arkansas, United States; University of Arkansas for Medical Sciences, Little Rock, Arkansas, United States; University of Arkansas for Medical Sciences, Little Rock, Arkansas, United States; University of Arkansas for Medical Sciences, Litttle Rock, Arkansas, United States; University of Arkansas for Medical Sciences, Little Rock, Arkansas, United States; University of Arkansas for Medical Sciences, Little Rock, Arkansas, United States

## Abstract

Arkansas has the second highest legal opioid prescribing rate in the U.S. We did a pilot study to assess the feasibility of using tablets to measure chronic pain and its impact of cognition, sleep, depression, and quality of life in older adults. Seventy-one older adults were trained in the use of tablets to use at home for 90 days that were pre-loaded with surveys on opioids, daily pain diary, sleep Pittsburgh Sleep Quality Index (PSQI), and Geriatric Depression scale (GDS-15). 1,944 responses were recorded over three months by 71 participants with mean age ± SD 70 ± 8 years (range 55-91). Males 23%, females 50%. Of females, 51% were African American and 49% white. All but one male was white. 80% of participants reported some pain with most ratings between 5 or 6 out of 10 (10 being worst). None of the participants were on opioids during the period of the study. Memory symptoms were reported most frequently in 28% of respondents, followed by daytime sleepiness in 33%. PSQI scores indicated poor sleep quality in 28%. Only one participant’s GDS score suggested depression. The feasibility of using tablet-based assays in older adults living in rural regions was successfully demonstrated, showing that older adults are receptive to learning new information and recording their health parameters on a tablet. The data obtained can provide clinicians a broader picture of the older adults pain-associated symptomatology and thereby assist them in designing better personalized pain management programs.

